# N348I in the Connection Domain of HIV-1 Reverse Transcriptase Confers Zidovudine and Nevirapine Resistance

**DOI:** 10.1371/journal.pmed.0040335

**Published:** 2007-12-01

**Authors:** Soo-Huey Yap, Chih-Wei Sheen, Jonathan Fahey, Mark Zanin, David Tyssen, Viviane Dias Lima, Brian Wynhoven, Michael Kuiper, Nicolas Sluis-Cremer, P. Richard Harrigan, Gilda Tachedjian

**Affiliations:** 1 Molecular Interactions Group, Macfarlane Burnet Institute for Medical Research and Public Health, Melbourne, Victoria, Australia; 2 School of Applied Sciences and Engineering, Monash University, Churchill, Victoria, Australia; 3 Division of Infectious Diseases, University of Pittsburgh School of Medicine, Pittsburgh, Pennsylvania, United States of America; 4 Department of Microbiology, Monash University, Clayton, Victoria, Australia; 5 British Columbia Centre for Excellence in HIV/AIDS, Vancouver, British Columbia, Canada; 6 Victorian Partnership for Advanced Computing, Carlton South, Victoria, Australia; 7 Department of Medicine, Monash University, Melbourne, Victoria, Australia; San Diego VA Research Center for AIDS and HIV Infection, United States of America

## Abstract

**Background:**

The catalytically active 66-kDa subunit of the human immunodeficiency virus type 1 (HIV-1) reverse transcriptase (RT) consists of DNA polymerase, connection, and ribonuclease H (RNase H) domains. Almost all known RT inhibitor resistance mutations identified to date map to the polymerase domain of the enzyme. However, the connection and RNase H domains are not routinely analysed in clinical samples and none of the genotyping assays available for patient management sequence the entire RT coding region. The British Columbia Centre for Excellence in HIV/AIDS (the Centre) genotypes clinical isolates up to codon 400 in RT, and our retrospective statistical analyses of the Centre's database have identified an N348I mutation in the RT connection domain in treatment-experienced individuals. The objective of this multidisciplinary study was to establish the in vivo relevance of this mutation and its role in drug resistance.

**Methods and Findings:**

The prevalence of N348I in clinical isolates, the time taken for it to emerge under selective drug pressure, and its association with changes in viral load, specific drug treatment, and known drug resistance mutations was analysed from genotypes, viral loads, and treatment histories from the Centre's database. N348I increased in prevalence from below 1% in 368 treatment-naïve individuals to 12.1% in 1,009 treatment-experienced patients (*p* = 7.7 × 10^−12^). N348I appeared early in therapy and was highly associated with thymidine analogue mutations (TAMs) M41L and T215Y/F (*p* < 0.001), the lamivudine resistance mutations M184V/I (*p* < 0.001), and non-nucleoside RTI (NNRTI) resistance mutations K103N and Y181C/I (*p* < 0.001). The association with TAMs and NNRTI resistance mutations was consistent with the selection of N348I in patients treated with regimens that included both zidovudine and nevirapine (odds ratio 2.62, 95% confidence interval 1.43–4.81). The appearance of N348I was associated with a significant increase in viral load (*p* < 0.001), which was as large as the viral load increases observed for any of the TAMs. However, this analysis did not account for the simultaneous selection of other RT or protease inhibitor resistance mutations on viral load. To delineate the role of this mutation in RT inhibitor resistance, N348I was introduced into HIV-1 molecular clones containing different genetic backbones. N348I decreased zidovudine susceptibility 2- to 4-fold in the context of wild-type HIV-1 or when combined with TAMs. N348I also decreased susceptibility to nevirapine (7.4-fold) and efavirenz (2.5-fold) and significantly potentiated resistance to these drugs when combined with K103N. Biochemical analyses of recombinant RT containing N348I provide supporting evidence for the role of this mutation in zidovudine and NNRTI resistance and give some insight into the molecular mechanism of resistance.

**Conclusions:**

This study provides the first in vivo evidence that treatment with RT inhibitors can select a mutation (i.e., N348I) outside the polymerase domain of the HIV-1 RT that confers dual-class resistance. Its emergence, which can happen early during therapy, may significantly impact on a patient's response to antiretroviral therapies containing zidovudine and nevirapine. This study also provides compelling evidence for investigating the role of other mutations in the connection and RNase H domains in virological failure.

## Abstract 2

Analyzing HIV sequences from a Canadian cohort, Gilda Tachedjian and colleagues identify a common mutation in a little-studied domain of reverse transcriptase that confers resistance to two drug classes.

## Editors' Summary

**Background.:**

In the 1980s, infection with the human immunodeficiency virus (HIV), which causes acquired immunodeficiency syndrome (AIDS), was a death sentence. Although the first antiretroviral drugs (compounds that block HIV's life cycle) were developed quickly, single antiretrovirals only transiently suppress HIV infection. HIV rapidly accumulates random changes (mutations) in its genetic material, some of which make it drug resistant. Nowadays, there are many different antiretrovirals. Some inhibit the viral protease, an enzyme used to assemble new viruses. Others block reverse transcriptase (RT), which makes replicates of the genes of the virus. Nucleoside/nucleotide RT inhibitors (NRTIs; for example, zidovudine—also called AZT—and lamivudine) and non-nucleoside RT inhibitors (NNRTIs; for example, nevirapine and efavirenz) interfere with the activity of RT by binding to different sites in its so-called “DNA polymerase domain,” the part of the enzyme that constructs copies of the viral genes. Highly active antiretroviral therapy (HAART), which was introduced in the mid 1990s, combines several antiretrovirals (usually a protease inhibitor and two NRTIs or an NNRTI and two NRTIs) so that the replication of any virus that develops resistance to one drug is inhibited by the other drugs in the mix. When treated with HAART, HIV infection is usually a chronic, stable condition rather than a fatal disease.

**Why Was This Study Done?:**

Unfortunately, HIV that is resistant to drugs still develops in some patients. To improve the prevention and management of drug resistance, a better understanding of the mutations that cause resistance is needed. Resistance to RT inhibitors usually involves mutations in the DNA polymerase domain that reduce the efficacy of NRTIs (including thymidine analogue mutations—also known as TAMs—and lamivudine-resistance mutations) and NNRTIs. Blood tests that detect these resistance mutations (genotype tests) have been used for several years to guide individualized selection of HIV drugs. Recently, however, mutations outside the DNA polymerase domain have also been implicated in resistance to RT inhibitors. In this study, the researchers have used data and samples collected since the mid 1990s by Canada's British Columbia Centre for Excellence in HIV/AIDS to investigate the clinical relevance of a mutation called N348I. This mutation changes an asparagine (a type of amino acid) to an isoleucine in a region of RT known as the connection domain. The researchers have also investigated how this mutation causes resistance to RT inhibitors in laboratory tests.

**What Did the Researchers Do and Find?:**

The researchers analyzed the first two-thirds of the RT gene in viruses isolated from a large number of the Centre's patients. Virus carrying the N348I mutation was present in less than one in 100 patients whose HIV infection had never been treated, but in more than one in 10 treatment-experienced patients. The mutation appeared early in therapy, often in viruses that had TAMs, a lamivudine-resistance mutation called M184V/I, and/or NNRTI resistance mutations. Patients treated with zidovudine and nevirapine were 2.6 times more likely to have the N348I mutation than patients not treated with these drugs. Furthermore, the appearance of the N348I mutation often coincided with an increase in viral load, although other mutations that appeared at a similar time could have contributed to this increase. When the researchers introduced the N348I mutation into HIV growing in the laboratory, they found that it decreased the susceptibility of the virus to zidovudine and to NNRTIs.

**What Do These Findings Mean?:**

These findings show that the treatment of patients with RT inhibitors can select a drug-resistant HIV variant that has a mutation outside the enzyme's DNA polymerase domain. Because this N348I mutation, which is commonly selected in vivo and has also been seen in other studies, confers resistance to two classes of RT inhibitors and can emerge early during therapy, it could have a large impact on patient responses to antiviral regimens that contain zidovudine and nevirapine. Although these findings do not show that the N348I mutation alone causes treatment failure, they may have implications for genotypic and phenotypic resistance testing, which is often used to guide treatment decisions. At present, genotype tests for resistance to RT inhibitors look for mutations only in the DNA polymerase domain of RT. This study is the first to demonstrate that it might be worth looking for the N348I mutation (and for other mutations outside the DNA polymerase domain) to improve the ability of genotypic and phenotypic resistance tests to predict treatment outcomes.

**Additional Information.:**

Please access these Web sites via the online version of this summary at http://dx.doi.org/10.1371/journal.pmed.0040335.

- Information is available from the US National Institute of Allergy and Infectious Diseases on HIV infection and AIDS
- HIV InSite has comprehensive information on all aspects of HIV/AIDS, including links to fact sheets (in English, French, and Spanish) about antiretrovirals, and chapters explaining antiretroviral resistance testing
- NAM, a UK registered charity, provides information about all aspects of HIV and AIDS, including fact sheets on types of HIV drugs, drug resistance, and resistance tests (in English, Spanish, French, Portuguese, and Russian)
- The US Centers for Disease Control and Prevention provides information on HIV/AIDS and on treatment (in English and Spanish)
- AIDSinfo, a service of the US Department of Health and Human Services provides information for patients on HIV and its treatment

## Introduction

The advent of highly active antiretroviral therapy has dramatically improved the clinical status of many HIV-infected patients. However, one of the major contributing factors to virological failure during highly active antiretroviral therapy is the selection and evolution of drug-resistant HIV strains [1,2]. Accordingly, a better understanding of drug resistance is needed to effectively prevent and manage resistance.

HIV-1 reverse transcriptase (RT) catalyses the conversion of the viral single-stranded genomic RNA into a double-stranded proviral DNA precursor. Due to its essential role in HIV replication, RT is a major target for chemotherapeutic intervention. In this regard, 11 of the 24 anti–HIV-1 inhibitors approved by the US Food and Drug Administration are RT inhibitors (RTIs). These can be divided into two therapeutic classes: (i) the nucleoside/nucleotide RT inhibitors (NRTIs), such as zidovudine (AZT) and lamivudine (3TC), that bind in the active site of the RT and act as competitive chain terminating inhibitors of DNA polymerisation [3]; and the non-nucleoside RT inhibitors (NNRTIs), such as nevirapine (NVP) and efavirenz (EFV), that bind to a nonactive site pocket in the HIV-1 RT (termed NNRTI-binding pocket) and act as allosteric inhibitors of DNA polymerisation [4–6].

HIV-1 RT is an asymmetric dimer that consists of a 66-kDa (p66) subunit and a p66-derived 51-kDa (p51) subunit. The catalytically active p66 subunit comprises the DNA polymerase (residues 1–315), connection (residues 316–437), and ribonuclease H (RNase H; residues 438–560) domains [7,8]. The polymerase domain can be further divided into the fingers, palm, and thumb subdomains [7,8]. Almost all RTI resistance mutations identified to date map to the DNA polymerase domain of RT, although this may be due in part to the fact that the connection and RNase H domains are not usually analysed in clinical samples [2,9]. In fact, none of the genotyping or phenotyping assays currently available for patient management assay the entire RT coding region, and genotyping algorithms used to predict drug resistance are generally confined to the analysis of mutations in the first 240 amino acids of the RT [9].

Recently, however, a growing body of evidence has emerged that implicates mutations outside of the DNA polymerase domain in RTI resistance. These include the G333D/E polymorphism that facilitates dual AZT/3TC resistance in viruses that contain both thymidine analogue mutations (TAMs) and M184V [10,11] and the Y318F mutation that confers NNRTI resistance [12]. A more recent study that performed genotypic and phenotypic analyses from a small number of NRTI-experienced and treatment-naïve individuals demonstrated that the C-terminal domains obtained from treatment-naïve patients did not increase AZT resistance compared to wild-type (WT) virus [13]. By contrast the same domains obtained from treatment-experienced patients conferred increased AZT resistance in the context of a WT polymerase domain by 3.6- to 5.8-fold and by 125- to 536-fold in the context of a polymerase domain encoding TAMs. Mutational studies revealed the presence of several connection domain mutations, including E312Q, G335C/D, N348I, A360I/V, V365I, and A376S, that were associated with AZT resistance [13]. In addition to changes in the connection domain, the mutations H539N and D549N in the RNase H domain have been shown to increase AZT resistance in a WT genetic background and in combination with TAMs [14]. Finally, a recent study reported that A371V in the connection domain and Q509L in the RNase H domain of HIV-1 RT were selected in combination with TAMs when virus was passaged under increasing concentrations of AZT [15]. Together, these mutations enhance AZT resistance in the presence of TAMs [15]. However, the in vivo significance of the above-mentioned studies is unknown.

In this study, we evaluated the in vivo relevance and role in drug resistance of a common RT connection domain mutation, N348I, using a variety of complementary approaches. The prevalence of N348I was evaluated in treated versus therapy-naïve individuals; its order of appearance relative to other drug resistance mutations was determined; and its association with virological failure was compared with other TAMs. The impact of N348I on decreased susceptibility to AZT and NNRTIs was determined in cell culture-based assays and further confirmed in biochemical studies with recombinant RT.

## Methods

### The British Columbia Centre for Excellence in HIV/AIDS Drug Treatment Program

Plasma samples and clinical data were obtained from the British Columbia Centre for Excellence in HIV/AIDS (the Centre), which has been previously described in detail [16]. The Centre has been responsible for the distribution and the population-based monitoring of antiretroviral treatment in British Columbia since 1992. The majority of HIV-1–positive men and women who have been treated are from Vancouver and the surrounding region. Since 1986, over 7,000 HIV-1–infected individuals have received antiretroviral treatment. The Centre distributes antiretroviral drugs based on guidelines generated by the Therapeutic Guidelines Committee, whose membership includes physicians, virologists, pharmacists, health service researchers, and economists. The Centre's HIV/AIDS drug treatment program has received ethical approval from the University of British Columbia Ethics Review Committee at its St. Paul's Hospital site. The program also conforms to the province's Freedom of Information and Protection of Privacy Act.

### Data Collection

A detailed description of the collection method and monitoring of data for HIV-1–positive individuals receiving antiretroviral therapy in the Centre was published previously [16]. The Centre recommends that viral load and CD4 cell counts be monitored at baseline, at 4 wk after initiation of antiretroviral therapy, and every 3 mo thereafter. Plasma samples were stored and frozen at −20 °C. Viral load was determined using the Roche Amplicor Monitor Assay (Roche Diagnostics, Laval, Quebec, Canada) using either the standard or ultrasensitive method (since 1999). CD4 counts were measured by flow cytometry, followed by fluorescent monoclonal antibody analysis (Beckman Coulter, Mississauga, Ontario, Canada). The Centre's HIV genotypic drug resistance database consists of over 25,000 samples. Genotypic resistance testing was performed on stored plasma HIV RNA samples [17–19]. HIV RNA extraction, RT-PCR amplification, and DNA sequencing were performed as previously described [16]. Results of the genotyping analysis were reported as amino acid changes in the HIV-1 RT with respect to a WT reference sequence (HIV-1 HXB2).

### Prevalence of Mutations from Codons 240 to 400 in Drug-Naïve and Experienced Individuals

Samples were analysed from individuals participating in the HIV/AIDS Drug Treatment Program from 1996 to 2003. The analysis was restricted to patient samples where sequencing was performed up to codon 400 of the RT gene. The latest available “on-therapy” sample was analysed for the treatment-experienced individuals, while a baseline sample, taken prior to therapy, was used for the therapy-naive individuals. The treatment-experienced dataset was randomly divided into two groups: a discovery test set of 500 individuals and an independent validation set of 509 individuals. Samples from 368 therapy naïve individuals were analysed to determine the prevalence of mutations in the absence of antiretroviral treatment.

### Association of N348I with Key Drug Resistance Mutations

The last on-therapy sample from a total of 3,569 patients was analysed for the association of N348I with key drug resistance mutations (*n* = 161) compared to the prevalence of key mutations in the absence of N348I (*n* = 3,408). Key drug resistance mutations were defined as those listed by the International AIDS Society-USA (IAS-USA) Drug Resistance Mutations Group [9]. Only one sample was analysed for each patient, and the samples that contained mixtures of WT and mutant were treated as mutant.

### Analysis of Drug Treatment Associated with Emergence of N348I

The following baseline predictor variables were investigated: age; gender; CD4 cell count; log_10_ transformed viral load (pVL); prior exposure to NRTIs, NNRTIs, or protease inhibitors (PIs); physician experience (per 100 patients); an AIDS diagnosis; history of injection drug use; year of first therapy; and adherence. The analysis was restricted to patients who were antiretroviral therapy naïve at baseline. Estimates of adherence to antiretroviral therapy were based on medications actually dispensed, not prescribed. For this study, we limited our measure of adherence to the first year of therapy estimated by dividing the number of months of medications dispensed by the number of months of follow-up. This measure of adherence has been found to be independently associated with HIV-1 viral suppression and survival amongst HIV-1-infected persons enrolled in the HIV/AIDS Drug Treatment Program [20,21]. Adherence was categorised as 0% to <40%, 40% to <80%, 80% to <95%, and ≥95%. To determine which antiretroviral drug treatment was associated with the emergence of N348I, an explanatory logistic regression model was developed for identifying which patient characteristics were most influential in the emergence of N348I during antiretroviral therapy. A backward stepwise technique was used in the selection of covariates. The selection of variables was based on two criteria: Akaike Information Criterion (AIC) and Type III *p*-values. These two criteria balance the model choice on finding the best explanatory model (Type III *p*-values: lower *p*-values indicate more significance) and at the same time a model with the best goodness-of-fit statistic (AIC: lower values indicate better fit). AIC is a criterion that penalised larger models, with equal fit. It can be used to compare models of different size, provided they are nested within each other. At each step of this process, the AIC value and the Type III *p*-values of each variable are recorded. Also at each step, the variable with the highest Type III *p*-value is dropped until there are no more variables left in the model. The final model is the model with the lowest AIC. The area under the ROC (receiver operating characteristic) curve was used to measure the model's ability to discriminate between those who developed resistance and those who did not [22].

### Pattern of Appearance of N348I Early in Drug Therapy

This analysis was performed on a total of 31 individuals who received treatment between June 1996 and February 2006. Neither N348I nor other key drug resistance mutations, as defined by the IAS-USA guidelines [9], were detected in the viral genomes at the start of antiretroviral therapy. The time in days after initiation of antiretroviral therapy of the first appearance of N348I and key drug resistance mutations was determined. Samples that contained mixtures of both WT and mutant at key codons were considered mutant. Where N348I appeared at a time point immediately following its first appearance, any new key mutations, which had not appeared previously, were also recorded.

### Association of N348I and TAMs with Changes in Viral Load

This analysis was performed on data from 7,074 patients from the BC Centre database between June 1996 and June 2007 where the change in viral load (ΔpVL) was defined as pVL at the time of the first positive test for the mutation subtracted by the pVL immediately before the first positive test for the mutation. The analysis was performed for N348I and for each of the TAMs: M41L, D67N, K70R, L210W, T215Y/F, and K219Q/E. The analysis excluded sequencing data that were not performed up to codon 400 and did not account for other IAS-USA defined RT or PI drug resistance mutations that may have been selected with N348I or TAMs. Mixtures at each codon were considered positive for the mutation. An upper (100,000 copies/ml) and a lower (500 copes/ml) cutoff for viral load were introduced into the analysis to avoid introducing bias due to differences in samples analysed using the original viral load assay (range 500 to 750,000 copies/ml) and the ultrasensitive viral load assay (range 50 to 100,000 copies/ml).

### Drugs and Reagents

The RT inhibitors AZT, 3TC, NVP, and EFV were obtained from the AIDS Research and Reference Reagent Program, National Institute for Allergy and Infectious Diseases, National Institutes of Health (NIH). 3′-Azido-2′,3′-dideoxythymidine triphosphate (AZT-TP) was purchased from TriLink Biotechnologies. All other nucleotides were purchased from GE Healthcare.

### Cells

MT-2 cells [23] were cultured in RF-10 buffered with 25 mM HEPES as previously described [24]. The TZM-bl indicator cell line [25], obtained through the NIH AIDS Research and Reference Reagent Program, and 293T cells were cultured in DMEM-10 [26].

### HIV-1 Infectious Molecular Clones and Site-Directed Mutagenesis

The pDRNLXN construct (a gift from Johnson Mak) was derived from the NL4.3 infectious molecular clone of HIV-1 [27] and was engineered with silent mutations that introduce XbaI and NotI restrictions sites at nucleotides 2319 and 3938, respectively. The pSVC21 construct contains the infectious HXB-2 molecular clone of HIV-1 [28]. HIV-1 clones, containing mutations in the RT coding region, were constructed by site-directed mutagenesis. Briefly, pSK-Bluescript II (Stratagene) with a 4.32-kb ApaI-SalI insert expressing the RT coding region from pDRNLXN or pSVC21 was modified by site-directed mutagenesis using either the QuikChange II Site Directed or QuikChange Multi Site Directed Mutagenesis kits (Stratagene) according to manufacturer's instructions. For mutations in the pNL4.3 backbone, full-length mutant infectious molecular clones of HIV-1 were constructed by replacement of the XbaI-NotI fragment in WT pDRNLXN with the modified XbaI-NotI fragment obtained by site-directed mutagenesis. For pSVC21, the WT ApaI-SalI insert was replaced with the same insert containing the desired mutations(s). All HIV-1 constructs were verified by nucleotide sequencing.

### Generation of HIV-1 Stocks and Titration

HIV-1 virus stocks were generated by transfecting 239T cells with infectious molecular clones of HIV-1 using the calcium phosphate technique as previously described [29]. The resulting virus was concentrated by ultracentrifugation through a 25% w/v sucrose cushion at 100,000*g* for 1 h at 4 °C. The 50% tissue culture infective dose (TCID_50_) was determined by endpoint titration in MT-2 cells as previously described [30] for HIV-1 stocks that were tested in drug susceptibility assays performed in MT-2 cells. The virus titre (in blue foci-forming units) was determined in TZM-bl cells as described previously [29] for HIV-1 stocks that were tested in drug susceptibility assays performed in TZM-bl cells.

### Phenotypic Drug Susceptibility Assays

Drug susceptibility assays were performed in either MT-2 cells using cell viability as the readout [24,31] or TZM-bl cells using the production of blue foci-forming cells as the readout. In assays performed in MT-2 cells, 100 TCID_50_ of appropriate virus was used to infect 6,000 cells in quadruplicate wells of a 96-well plate. After 5- to 6-d incubation at 37 C and 5% CO_2_, cell viability, which was used to measure the inhibitory effects of the drugs on virus-specific cytopathic effects, was determined using the CellTiter 96 Aqueous One Solution Cell Proliferation Assay according to manufacturer's instructions (Promega). For assays performed in the TZM-bl indicator cell line, cells were seeded at 2 × 10^4^ per well of a 96-well plate 24 h before infection. At 1 to 2 h prior to infection, cultures were replaced with fresh DMEM-10 with or without the appropriate RTI. Assays were performed in quadruplicate wells. Virus was added to cells in the presence of 10 μg/ml DEAE-dextran at an inoculum of 50–150 blue foci-forming units per well. At 12–13 h postinfection, cells were replaced with fresh DMEM-10 containing drug at the appropriate concentration. After 48 h postinfection, cultures were fixed and stained for β-galactosidase activity as previously described [29]. Assays were blinded and blue foci-forming units were counted on an inverted microscope. For each virus, the 50% inhibitory concentration (IC_50_) was determined by the “sigmoidal dose-response, variable slope” nonlinear regression analysis of the percentage inhibition of virus versus the log_10_ concentration of inhibitor using GraphPad Prism for Windows (version 4.03) software (GraphPad Software). Percentage inhibition of virus was calculated at each drug concentration for assays performed in MT-2 cells as previously described [31]. The percentage inhibition of HIV-1 replication at a given drug concentration in assays performed in TZM-bl cells was calculated using the formula [(*x* – *y*)/*x*] × 100, where *x* is the mean blue foci-forming units from four virus-only control wells and *y* is the mean blue foci-forming units from four HIV-1–infected wells at a given drug concentration.

### Expression, Purification, and Characterisation of Recombinant HIV-1 RT

The M41L, L210W, T215Y, and N348I mutations were introduced into WT HIV-1_LAI_ RT [32] by site-directed mutagenesis using the QuikChange Mutagenesis kit. Full-length sequencing of mutant RTs was performed to confirm the presence of the desired mutations. Recombinant WT and mutant HIV-1 RT was overexpressed and purified to homogeneity [33,34], and the enzyme's active site concentrations were determined as described previously [35]. All enzymes were found to exhibit similar levels of DNA polymerisation activity (unpublished data). The sensitivity of the WT and mutant enzymes to AZT-TP, NVP, and EFV inhibition were determined under steady-state assay conditions, as described previously [36,37].

### Polyacrylamide Gel Electrophoresis Analysis of RT Polymerisation Products Formed under Continuous DNA Polymerisation Conditions

Heteropolymeric RNA-dependent or DNA-dependent DNA polymerase template/primer (T/P) were prepared as described previously [38]. DNA polymerisation reactions were carried out by incubating 200 nM WT or mutant RT with 20 nM heteropolymeric T/P complex in 50 mM Tris-HCl (pH 8.0) and 50 mM KCl for 5 min before the addition of 5 μM of each dNTP, 2.5 μM AZT-TP, 3.0 mM ATP, and 10 mM MgCl_2_. After defined incubation periods, aliquots were removed from the reaction tube and quenched with equal volumes of gel loading dye. Products were separated by denaturing gel electrophoresis and quantified with a Bio-Rad GS525 Molecular Imager.

### Assay of RT-Catalysed Phosphorolysis of AZT-Monophosphate Terminated Primers

A 5′ ^32^P-labeled 26-nucleotide primer (5′-CCTGTTCGGGCGCCACTGCTAGAGAT-3′) annealed to a 35-nucleotide DNA template (5′-AGAATGGAAAATCTCTAGCAGTGGCGCCCGAACAG-3′) or RNA template (5′-AGAAUGGAAAAUCUCUAGCAGUGGCGCCCGAACAG-3′) was chain-terminated with AZT-monophosphate (AZT-MP), as described previously [39,40]. The phosphorolytic removal of AZT-MP was achieved by incubating 200 nM active site RT with 20 nM chain-terminated T/P complex in 50 mM Tris-HCl (pH 8.0) and 50 mM KCl. The reaction was initiated by the addition of 3.0 mM ATP, 1 μM TTP, 5 μM ddCTP, and 10 mM MgCl_2_. After defined incubation periods, aliquots were removed from the reaction tube and processed as described above.

### Assay for RT RNase H activity

WT and mutant RT RNase H activity was evaluated using the same AZT-MP chain-terminated RNA/DNA T/P substrate described above, except the 5′-end of the RNA was ^32^P-end-labeled. Assays were carried out using 20 nM T/P, 3 mM ATP, and 10 mM MgCl_2_ in a buffer containing 50 mM Tris-Cl (pH 8.0) and 50 mM KCl. Reactions were initiated by the addition of 200 nM WT or mutant HIV-1 RT. Aliquots were removed, quenched at varying times, and analysed as described above.

### Statistical Analyses

Statistical analyses of the prevalence of mutations from codons 240 to 400 in drug-naïve compared to drug-treated individuals and the association of N348I with key drug resistance mutations were performed using the Chi-square test. In the descriptive analysis used to determine which antiretroviral drug treatment was associated with the emergence of N348I, categorical variables were compared using the Chi-square or Fisher Exact test, and continuous variables were compared using the Wilcoxon rank-sum test. In the case of multiple comparisons, the Bonferroni and/or the Benjamini and Hochberg methods were used [41–45], although only the most significant values are reported here. For the analysis of the association of N348I and TAMs with changes in viral load, the ΔpVL values were assessed for normality as determined visually by normal probability plots and by the one-sample Kolmogorov-Smirnov goodness-of-fit test. The one-sample *t*-test was used to test the alternative hypothesis of the ΔpVL being equal to or less than 0 if ΔpVL was normally distributed. If not normal, the ΔpVL values were tested using the Wilcoxon rank-sum test. The statistical significance of differences between IC_50_ values obtained in cell culture drug susceptibility assays was determined using the Wilcoxon rank-sum test [46].

## Results

### N348I Is Prevalent in Patients after Antiretroviral Therapy

The prevalence of mutations at HIV RT codons 1–400 observed by population sequencing analysis of plasma-derived HIV from individuals with known treatment histories (*n* = 1,009) was compared with their prevalence in samples from therapy-naïve individuals (*n* = 368). The treatment-experienced dataset was divided randomly into two groups: a discovery test set of 500 individuals and an independent validation set of 509 individuals. N348I, a mutation located in the connection domain of the RT, increased in prevalence from below 1% in samples from untreated individuals to greater than 12% in the discovery test and independent validation subsets of samples from treated individuals (*p* = 7.7 × 10^−12^) (Table 1). N348I was ranked as the ninth most prevalent mutation of a total of 39 RT codons evaluated and was more common than several “key” RTI mutations that are used in genotypic resistance analyses; i.e. mutations at codons 210, 190, and 74 were ranked tenth, 16^th^, and 18th, respectively (unpublished data). Several other mutations increased in prevalence significantly in samples from RTI-experienced individuals after adjustment for multiple comparisons using either the Bonferroni or Benjamini and Hochberg methods. These include previously described novel mutations in the polymerase domain [47] and mutations in the RT connection domain at codons G359, A371, and K356 (ranked 13th, 20th and 27th, respectively). However, because N348I was not polymorphic and was also the most prevalent mutation in the RT connection domain, we concentrated on characterising its role in RTI drug resistance.

**Table 1:**
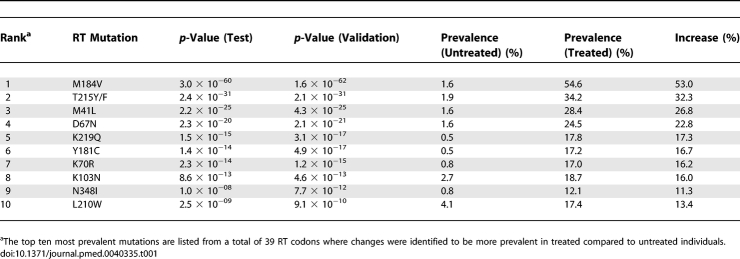
Prevalence of RT Mutations in Treated and Untreated Patients

### Association of N348I with Known RT Drug Resistance Mutations

**Figure 1:**
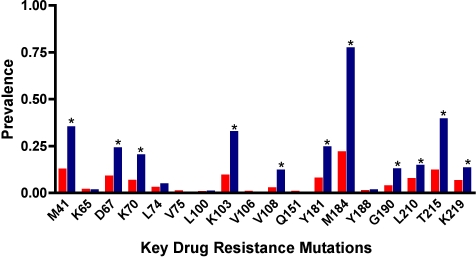
Association of N348I with Key Drug Resistance Mutations The prevalence of key drug resistance mutations without N348I (red bars) compared to their prevalence with N348I (blue bars). The last on therapy sample from a total of 3,569 patients was analysed where 3,408 and 161 samples did not or did contain N348I, respectively. Statistically significant differences between the two groups were determined by Chi-square analysis. The asterisk above the bars denotes *p* < 0.001, except for L210 where *p* = 0.002.

To determine whether N348I was associated with the presence of other IAS-USA-defined RTI resistance mutations, we analysed the last on-therapy sample from 161 patients with N348I compared with 3,408 without N348I. N348I was highly associated with several key drug resistance mutations, including M184V/I (*p* = 6.6 × 10^−45^) the TAMs M41L (*p* = 1.8 × 10^−14^), D67N (*p* = 7.7 × 10^−10^), K70R (*p* = 1.4 × 10^−10^), T215Y/F (*p* = 6.0 × 10^−21^), K219Q/E (*p* = 8.0 × 10^−4^), and L210W (*p* = 0.002), and the NNRTI resistance mutations K103N (*p* = 1.1 × 10^−19^), V108l (*p* = 8.4 × 10^−13^), Y181C/I (*p* = 4.7 × 10^−13^) and G190A/S (*p* = 8.0 × 10^−9^) (Figure 1). While all TAMs were significantly associated with N348I, there was a stronger association with mutations at codons 41, 67, 70, and 215 that emerge early in drug therapy compared with mutations at codons 210 and 219 that tend to appear late in therapy [48].

### Association of N348I with Specific Drug Treatment

The association of N348I with several baseline predictor variables was analysed. Univariate analyses demonstrated a significant association with male gender (odds ratio [OR] 2.23, 95% confidence interval [CI] 1.21–4.13), increased adherence (OR 1.13, 95% CI 1.05–1.22), and increased physician's experience (OR 1.24, 95% CI 1.03–1.48), AZT treatment (OR 1.60, 95% CI 1.07–2.40), and NVP treatment (OR 1.53, 95% CI 1.02–2.29). The association of predictor variables with N348I was analysed by multivariate logistic regression analysis. These data demonstrate that treatment with AZT compared with treatment that did not consist of either AZT or NVP was associated with an increased risk for N348I, while there was no increased risk for N348I with NVP treatment compared with treatment with neither AZT nor NVP (Table 2). However, combined treatment with AZT and NVP was associated with a 2.62-fold increased risk in the emergence of N348I compared with treatment regimens containing neither drug (Table 2). Other variables associated with increased risk of emergence of N348I following multivariate analysis included increased adherence and physician's experience. In contrast, there was a negative association with N348I and treatment initiation taking place between 2000 and 2003 (OR 0.58, 95% CI 0.35–0.98) or between 2003 and 2005 (OR 0.27, 95% CI 0.10–0.78) relative to patients starting before 2000, perhaps due to the fact that prescription of combination therapy with AZT and NVP has been less frequent after the year 2000.

**Table 2:**
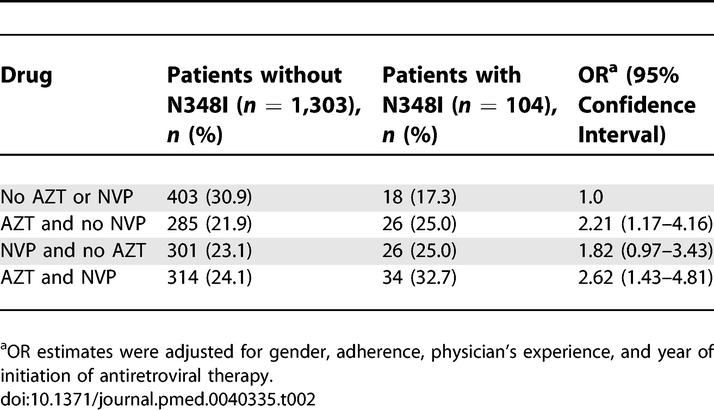
Association of N348I with Specific Drug Therapy

### Pattern of Emergence of N348I Early in Drug Therapy

**Figure 2:**
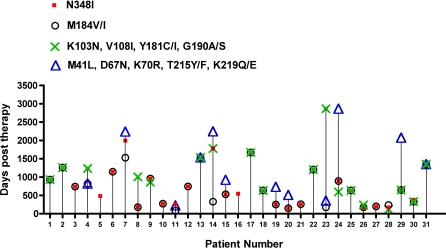
Pattern of Emergence of N348I Early in Drug Therapy Failure The pattern of emergence of N348I relative to key drug resistance mutations in the RT is shown with respect to days post therapy. These patients had neither N348I nor key drug resistance mutations present at baseline. Mixtures of WT and mutants were considered mutant in this analysis. The presence of the indicated key mutations was included from data available from each time point up to the appearance of N348I. If subsequent time points also contained N348I, then the key mutations present at this time point was also included in the analysis.

In order to determine whether N348I appears early in drug therapy and its pattern of emergence with respect to key drug resistance mutations, we performed an analysis on samples from a subset of patients in the Centre's database who had neither N348I nor other IAS-USA-resistance mutations present at baseline and for whom complete treatment history and viral data were known. Of the 31 patients fitting these criteria, we observed that N348I tended to appear in plasma virus relatively early after initiation of antiretroviral therapy. Moreover, the appearance of N348I was observed during virological failure (viral loads 283–591,000 copies/ml, median 3,600). In general, N348I appeared at the same time as M184V/I and before the appearance of TAMs (Figure 2). N348I also usually appeared at the same time as key NNRTI resistance mutations (Figure 2). Notably, N348I was the first mutation that was observed in two patients (patients 5 and 16, with viral loads of 1,120 and 591,000 copies/ml, respectively) indicating an association with virological failure.

### Appearance of N348I Is Associated with a Concomitant Increase in Viral Load at Least Equivalent to That Seen with TAMs

To determine the potential in vivo significance of N348I, we examined whether the appearance of N348I was associated with an increase in viral load and compared this with the changes in viral load associated with each of the other TAMs (Table 3). Our analysis demonstrated that the appearance of N348I was associated with a significant median increase in pVL of 0.23 log_10_ copies/ml (*p* < 0.001) and that this increase was at least as large as the ΔpVL associated with each of the individual TAMs: M41L, D67N, K70R, L210W, T215Y/F, and K219Q/E (Table 3). These data indicate that the appearance of N348I is associated with an increase in viral load that is at least as large as that observed with any of the IAS-USA-defined TAMs. However, this analysis could not exclude the contribution of key PI or RT mutations that might have appeared simultaneously with the specific mutations being evaluated, due to the large number of possible permutations.

**Table 3:**
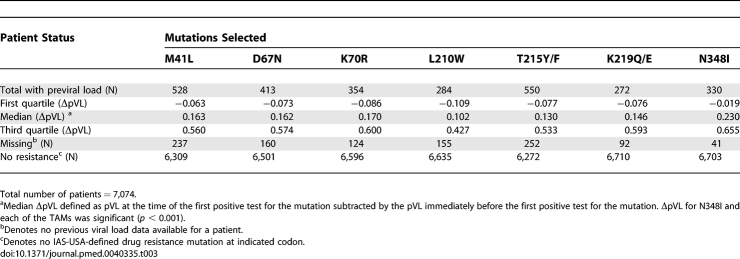
Appearance of N348I and TAMs Is Associated with an Increase in Viral Load

### N348I Confers Reduced Susceptibility to AZT in a WT Backbone and When Combined with TAMs

The impact of N348I on AZT susceptibility was examined by introducing this mutation by site-directed mutagenesis into the molecular clones HXB-2 (HX/348) and NL4.3 (NL/348) in order to evaluate the effect of N348I in two genetically distinct WT backbones. Phenotypic susceptibility assays were performed in MT-2 cells for the HXB-2 strain (HX) or in the TZM-bl indicator cell line for NL4.3 strain (NL). In comparison with the WT HIV-1, both the HX/348 (*p* = 0.05, *n* = 3) and NL/348 (*p* = 0.005, *n* = 8) mutant viruses demonstrated 2-fold decreased susceptibility to AZT (Table 4). In contrast, NL/348 showed no significant difference in 3TC susceptibility compared with WT NL (1-fold, *p* > 0.2, *n* = 5) (Table 4). Because N348I is highly associated with TAMs (Figure 1), we also introduced N348I into molecular clones that contained M41L and T215Y (NL/2AZT + 348) or M41L, L210W, and T215Y (HX/3AZT + 348) and assayed for AZT susceptibility (Table 4). NL/2AZT + 348 demonstrated a 2-fold decrease in AZT susceptibility compared with NL/2AZT (*p* = 0.047, *n* = 6) (Table 4), whereas HX/3AZT + 348 demonstrated a 4.0-fold decrease in AZT susceptibility compared with HX/3AZT (*p* = 0.05, *n* = 3). Taken together, these data demonstrate that N348I, alone and in combination with TAMs, confers AZT resistance.

**Table 4:**
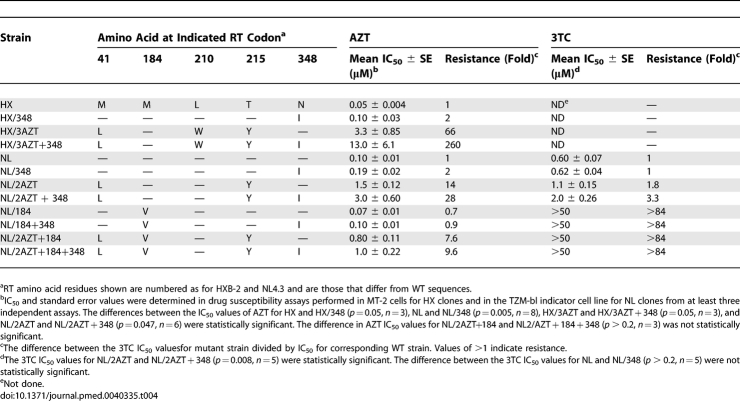
Effect of N348I on AZT and 3TC Resistance

### N348I Decreases 3TC Susceptibility in the Context of TAMs

Although N348I did not appear to confer 3TC resistance by itself, when combined with M41L and T215Y (NL/2AZT + 348), it conferred a 3.3-fold and 1.8-fold decrease in 3TC susceptibility compared to the WT (*p* = 0.005, *n* = 5) and NL/2AZT (*p* = 0.008, *n* = 5) viruses, respectively (Table 4). These data are consistent with a previous report demonstrating that TAMs confer decreased susceptibility to 3TC [49] and indicate that N348I further potentiates 3TC resistance in the context of TAMs. As expected, M184V conferred high-level resistance to 3TC. However, the impact of N348I on enhancing 3TC resistance in the context of M184V in either the absence or presence of the TAM combination M41L and T215Y could not be assessed as the 3TC IC_50_ values were beyond the detectable limits of our assay (Table 4). Nevertheless, any possible potentiating effect of N348I on 3TC resistance is unlikely to be clinically relevant given the high level of resistance conferred by M184V alone [50–52].

### N348I Does Not Counteract Antagonism between M184V and TAMs

M184V suppresses phenotypic resistance conferred by TAMs, although the effect decreases with an increase in the number of TAMs [53]. Because M184V appears with N348I and TAMs during virological failure (Figure 2), it is possible that N348I might be involved in facilitating dual AZT/3TC resistance in viruses that harbour both TAMs and M184V. Accordingly, we compared the AZT susceptibility of HIV-1 containing M184V and TAMs (M41L and T215Y) (NL/2AZT + 184) and HIV-1 containing M184V, TAMs, and N348I (NL/2AZT + 184 + 348). The data did not demonstrate a significant increase in the AZT IC_50_ for NL/2AZT + 184 + 348 compared with NL/2AZT + 184 (1.3-fold, *p* > 0.2, *n* = 3) (Table 4), suggesting that N348I does not counteract the antagonistic effects of M184V on phenotypic AZT resistance.

### N348I Confers Decreased Susceptibility to NNRTIs

Since N348I is also highly associated with key mutations that confer NNRTI resistance (Figures 1 and 2), we investigated whether N348I could decrease susceptibility to NVP and EFV. Remarkably, viruses harbouring N348I conferred significant decrease in NVP (7.4-fold) (*p* = 0.005, *n* = 5) and EFV (2.5-fold) (*p* = 0.005, *n* = 5) susceptibilities (Table 5). Similar to previous studies [54], K103N conferred resistance to both NVP (96-fold) and EFV (30-fold) (Table 5). Notably, when N348I was combined with K103N, we observed a significant potentiation of EFV resistance by N348I (7.5-fold) (*p* = 0.005, *n* = 5), resulting in HIV-1 that was highly resistant to EFV (226-fold) (Table 5). N348I also potentiated NVP resistance conferred by K103N, although we could not determine the exact level of enhancement as the IC_50_ value was greater than what could be measured in our assay. These data clearly demonstrate that N348I not only confers AZT resistance but also decreases susceptibility to NNRTIs. Furthermore, N348I also conferred EFV and NVP resistance at the enzyme level (Table 6).

**Table 5:**
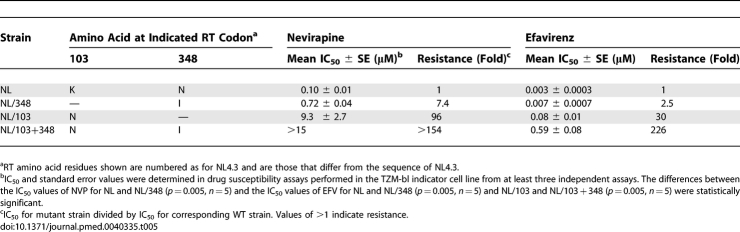
Effect of N348I on NNRTI Resistance

**Table 6:**
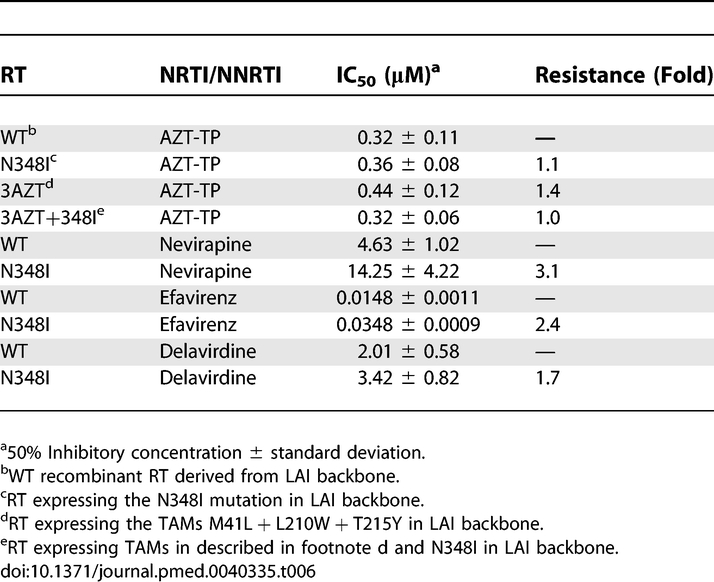
Drug Susceptibility of Recombinant RT to AZT-TP and NNRTIs

### Biochemical Mechanisms of AZT Resistance by Recombinant HIV-1 RT Containing N348I

NRTI-associated resistance mutations can be broadly categorised into two groups depending on their phenotypic mechanism of resistance [1]. TAMs augment the ability of HIV-1 RT to excise a chain-terminating NRTI-monophosphate from a prematurely terminated DNA chain [55–57]. In comparison, mutations such as K65R, K70E, L74V, Q151M, and M184V increase the selectivity of RT for incorporation of natural deoxynucleoside triphosphate substrate versus the NRTI-triphosphate [1]. To define the biochemical mechanism by which the N348I mutation in HIV-1 RT confers resistance to AZT, we designed and performed independent experiments that assessed whether N348I, alone or in combination with M41L, L210W, and T215Y (3AZT), conferred AZT resistance by a discrimination or excision phenotype.

**Figure 3:**
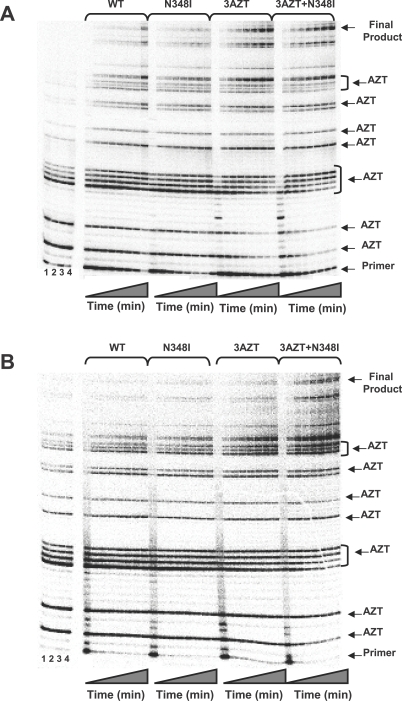
Steady-State DNA Synthesis by WT and Mutant HIV-1 RT in the Presence of AZT-TP and 3 mM ATP The experimental conditions for this experiment are described in Methods. (A) DNA synthesis was evaluated on a DNA/DNA T/P. (B) DNA synthesis was evaluated on an RNA/DNA T/P. For both (A) and (B), lanes demarcated as 1–4 included control reactions for the WT, N348I, 3AZT, and 3AZT + N348I RTs where all substituents were added except ATP. The primer, final product, and AZT-MP chain-termination sites are indicated.

Our data show that each of the four recombinant enzymes (WT, N348I, 3AZT, and 3AZT + N348I) was equally sensitive to inhibition by AZT-TP (Table 6), suggesting that N348I does not confer AZT resistance by a discrimination phenotype. Accordingly, the ability of the WT and mutant enzymes to excise AZT-MP from a chain-terminated primer was evaluated in two different experiments. In the first, we analysed steady-state synthesis by both WT and mutant enzymes in the presence of AZT-TP and ATP, using a heteropolymeric RNA or DNA template, corresponding to the HIV-1 sequence used for (–) strong stop DNA synthesis, primed with a DNA oligonucleotide [58]. The 173-nucleotide incorporation events needed to produce full-length DNA product in this assay system allow for multiple AZT-TP incorporation and AZT-MP excision events during the formation of full-length final product. Figure 3A shows that in the presence of 3 mM ATP, the 3AZT and 3AZT + N348I RTs synthesised significantly greater amounts of full-length DNA on the DNA/DNA T/P than either the WT or N348I enzymes. However, no significant differences were noted between the 3AZT and 3AZT+N348I enzymes (Figure 3A). By contrast, on the RNA/DNA T/P, the 3AZT + N348I enzyme synthesised greater amounts of full-length DNA product than any of the other enzymes (Figure 3B).

**Figure 4:**
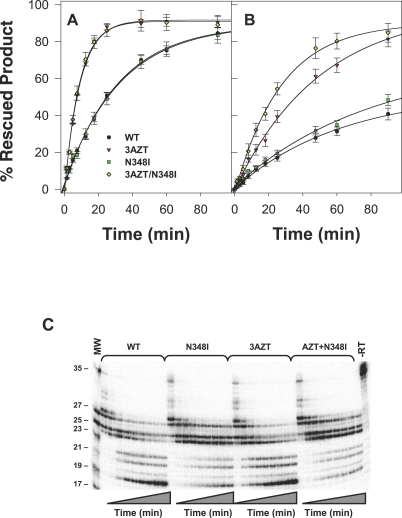
Isotherms for ATP-Mediated Phosphorolytic Excision of AZT-MP by WT, 3AZT, N348I, and 3AZT + N348I HIV-1 RT on DNA/DNA T/P (A) and RNA/DNA T/P (B). Data are the mean of three or four independent experiments. Autoradiogram of RNase H products generated during ATP-mediated excision assays on the RNA/DNA T/P (C). Experiments were carried out as described in Methods.

To further determine the role of N348I in the AZT excision phenotype, we next evaluated the ability of the WT or mutant RT to excise AZT-MP from the 3' terminus of the primer and to rescue DNA synthesis, as described previously [40,55]. These data show a similar trend to that observed in the steady-state assays (Figure 4A and 4B). The 3AZT and 3AZT + N348I RT were more efficient at excising AZT-MP and rescuing DNA synthesis than either the WT or N348I enzymes on a DNA/DNA T/P, but no significant differences were noted between the 3AZT and 3AZT/N348I enzymes (Figure 4A). However, on the RNA/DNA T/P, the 3AZT + N348I enzyme exhibited a greater capacity to excise AZT-MP than the 3AZT enzyme (Figure 4B). Taken together, these data suggest that the N348I mutation augments the AZT excision phenotype on an RNA/DNA T/P but not on a DNA/DNA T/P.

To further characterise this phenotype, we also assessed the RNase H cleavage events that occurred during the AZT-MP excision reactions described in the latter experiment. This analysis showed that the N348I mutation, alone and in combination with 3AZT, significantly decreased RNase H cleavage activity; and in particular, decreased formation of a secondary cleavage product that reduced the RNA template to 17 nucleotides (the corresponding RNA/DNA duplex length is reduced to ten nucleotides) (Figure 4C). The significance of this decreased RNase H cleavage activity is discussed later.

## Discussion

In this study, we demonstrate a role of the commonly selected N348I mutation in the connection domain of HIV-1 RT in both NRTI (AZT) and NNRTI (NVP and EFV) resistance. N348I is more prevalent in clinical samples from patients treated with RTIs compared with samples from treatment-naïve individuals. In this regard, our analysis ranks N348I as the ninth most prevalent resistance mutation from a total of 39 different RT codons that were evaluated in RTI experienced patients and this mutation was observed more frequently in our cohort than mutations at codons 210, 69, 44, 190, 118, and 74, most of which have been the topic of multiple clinical, genetic, virological, biochemical and structural analyses [59–63]. As expected with HIV, there was a strong codon bias, with most of the mutant N348I (98.6%) encoded by ATT, while more than 97% of WT N348 was encoded by AAT. Furthermore, the N348I mutation is associated with M184V/I, TAMs, K103N and Y181C and is selected by NVP and AZT treatment. The N348I mutation also appears relatively early in virological failure, generally before the appearance of TAMs and usually at the same time as the acquisition of NNRTI resistance mutations. Notably, N348I was the first mutation observed in two patients in our cohort. The early appearance of N348I after initiation of antiretroviral therapy is consistent with it playing a key role in the development of RTI resistance rather than being an accessory mutation that appears after primary mutations [64]. This is supported by our findings that its appearance is associated with an increase in viral load, which is at least as large as that observed for any of the other TAMs.

The high prevalence of N348I is not unique to the Centre's database as it was also identified in a separate analysis of mutations beyond RT codon 240 in a large US clinical database (Quest Diagnostics) [65]. In the latter study, two sets of sequences were analysed. The first comprised 41,122 de-identified clinical samples collected between January 2002 and June 2003 and the second comprised 16,449 samples collected between July 2003 and December 2003. Viruses with at least one resistance mutation in the protease or RT (64.8% in dataset 1 and 57% in dataset 2) were compared with those that contained no key drug resistance mutations (35.2%, dataset 1). The frequency of N348I in the first and second datasets was 13.6% and 13.0%, respectively. A recent study also demonstrates the presence of N348I in RT sequences from over 3,000 treated individuals with subtype B HIV-1 in a UK database [66]. Thus, the prevalence of N348I in three large databases indicates that this mutation is likely to be present in other clade B cohorts receiving antiretroviral therapy.

To delineate the role of N348I in RTI resistance, this mutation was introduced into molecular HIV-1 clones with defined genetic backbones. In this regard, N348I, alone or in combination with TAMs, conferred 2- to 4-fold AZT resistance. The level of AZT resistance conferred by N348I alone is comparable to the levels of resistance conferred by other individual TAMs. For example, the M41L mutation has been reported to confer between 1.4- to 4-fold AZT resistance, the K70R mutation up to 8-fold resistance, and T215Y up to 16-fold resistance [59,60,62,67,68]. Furthermore, other groups have also recently demonstrated that the N348I mutation confers both AZT and NNRTI resistance; with the Monogram group reporting a 2.4-fold change in AZT susceptibility [69], while another group reported a 23-fold change in AZT susceptibility [70]. In addition, a recent study demonstrated that reversion of N348I to the WT codon in an RT gene from an isolate derived from an RTI-experienced patient increased AZT sensitivity 5- to 10-fold [13]. Interestingly, N348I also conferred decreased susceptibility to EFV and NVP, data that are also consistent with studies from other groups [69,70]. In our cohort N348I was associated with the appearance of K103N, V108l, Y181C/I, and G190A/S early in virological failure (Figure 2) and was significantly associated with these mutations in analysis of the entire database (Figure 1). N348I significantly potentiated EFV and NVP resistance when combined with K103N. Consistent with our cell culture–based assays, NNRTI resistance was also observed at the enzyme level with recombinant RT expressing N348I. Furthermore, the phenotypic findings are consistent with our clinical data demonstrating the significant association of N348I with TAMs and NNRTI mutations and its selection in patients on AZT and NVP combination therapy.

N348I was also significantly associated with M184V/I in our cohort. N348I conferred a small increase in 3TC resistance in the context of TAMs but did not counteract the previously observed antagonism between M184V and TAMs. This is in contrast to another connection domain mutation, G333D/E, which is associated with dual resistance to AZT and 3TC [10]. It is curious that our data demonstrate a strong association of N348I with M184V/I despite it having little effect on drug susceptibility in the context of M184V. However, this can be explained by several possibilities, including that M184V/I and N348I are not genetically linked, that their coincident emergence is due to a high proportion of the Centre's cohort being prescribed 3TC, or that N348I has an effect on M184V/I that is independent of decreasing drug susceptibility such as altering viral fitness. Further studies will be needed to address these possibilities.

Apart from Q145M, which has been reported to confer broad resistance to several NRTIs and NNRTIs [71,72], and Y181C/I, which may confer resistance to stavudine in addition to NVP [73,74], N348I represents the only other example of an in vivo mutation that confers decreased susceptibility to two classes of RT inhibitors. However, in contrast to N348I, the prevalence of Q145M in an Italian cohort of 3,595 patients was lower (2.36%) and was always found in the presence of key drug resistance mutations [72]. In our cohort, Q145M was exceedingly rare with a prevalence of less than 0.5%. Additional studies also demonstrate that Q145M significantly decreases viral replication capacity and that the recombinant enzyme displays a large loss in catalytic efficiency [72]. While the effect of N348I on viral replication fitness has not been established, recombinant enzymes harbouring this mutation facilitate DNA synthesis reactions at least as efficiently as the WT enzyme (unpublished data).

**Figure 5:**
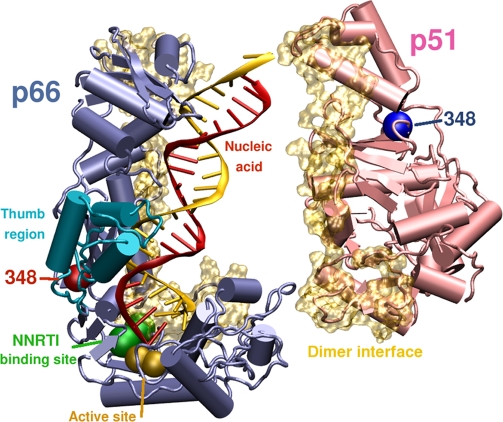
Position of N348 in the HIV-1 RT Structure Structural model of HIV-1 demonstrating the position of N348 in the p66 (blue) and p51 RT (pink) subunits relative to the polymerase active site (gold spheres), the NNRTI-binding pocket (green) and the dimer interface (gold). N348 in p66 (red sphere) is located in the connection domain and is in close proximity to the hinge region of the p66 thumb (turquoise). In contrast, N348 in p51 is located far from the dimer interface. The RT coordinates were derived from 1RTD [78], with C280 reverted to WT S280 and with missing residues of p51 added (amino acids 218 to 230 and 430 to 440), while the DNA/DNA duplex was converted to an RNA/DNA duplex. The image was prepared using POVRAY (Persistence of Vision Pty, Ltd, Williamstown, Victoria, Australia) and GNU Image Manipulation Program (GIMP) (http://www.gimp.org/).

Biochemical analyses designed to elucidate the mechanism by which N348I confers AZT resistance indicate that this mutation, consistent with other TAMs, acts by an excision rather than discrimination phenotype. However, an increase in AZT excision was only observed with a RNA/DNA T/P and not with a DNA/DNA T/P. Recently, it was suggested that mutations in the RT that decrease RNase H activity will enhance the AZT excision phenotype by slowing down the rate at which the RNA template strand is degraded [13,14]. In this regard, our analyses confirm that the N348I mutation, alone and in combination with TAMs, decreases the enzyme's RNase H activity. In particular, the mutation significantly reduces the appearance of a 17-nucleotide secondary cleavage product, which corresponds to RNA/DNA duplex length of ten nucleotides. Recent studies that were designed to delineate the relationship between T/P duplex length and efficiency of AZT excision demonstrated that RT could not efficiently unblock chain-terminated T/P if the RNA/DNA duplex length was less than 13 nucleotides [75]. Thus, the decrease in the formation of this cleavage product is significant and suggests a possible mechanism by which N348I confers AZT resistance. The structural mechanism by which N348I confers AZT (and NNRTI) resistance, however, cannot be inferred from this study. Recent studies have demonstrated that mutations in the RNase H primer grip region can significantly enhance AZT resistance by changing interactions between RT and the T/P complex and/or shifting the balance between the polymerase and RNase H activities [15,76]. In contrast, N348I does not make direct contacts with the T/P. Furthermore, N348 in p66 RT is 27 Å away from the polymerase active site and 20 Å from the NNRTI-binding pocket, while N348 in p51 RT is 60 Å from the NNRTI-binding pocket (Figure 5). Interestingly, N348 in p66 lies in close proximity to I270 and P272 at the base of the thumb domain in some crystal structures, suggesting a possible mechanism by which N348I mediates AZT resistance [77]. Nevertheless, additional biochemical and structural studies are warranted to define the exact mechanism by which N348I confers RTI resistance.

One of the major limitations of this study is that while the data demonstrate that the appearance of N348I is associated with an increase in viral load, which is at least as large as the recognised TAMs, it does not account for the simultaneous appearance of other key PI or RT drug resistance mutations that could also contribute to the observed increase in viral load. Nevertheless, this caveat also applies to our analysis of the individual TAMs. Even with this large study, the number of permutations of mutations is too large to be able to exclude the contribution of other mutations, particularly as these patients are on combination therapy. Nevertheless, this study represents, to our knowledge, the most thorough evaluation of the in vivo relevance of a drug resistance mutation that can contribute to HIV drug resistance, but is not presently included in most genotypic and phenotypic resistance assays.

In conclusion, we have demonstrated that a mutation in the connection domain of the HIV-1 RT, N348I, is prevalent in drug-treated individuals, appears relatively early in drug therapy, and confers decreased susceptibility to AZT and NNRTIs. The mechanism of AZT resistance can be ascribed to increased nucleotide excision, as observed from an RNA/DNA T/P, and is likely to be manifested through decreased RNase H activity conferred by N348I. Decreased susceptibility to NNRTIs can be demonstrated at the enzyme level. Taken together, our data underscore the important role of N348I in conferring drug resistance to AZT and NNRTIs.

## Supporting Information

### Accession Numbers

The GenBank (http://www.ncbi.nlm.nih.gov/Genbank) accession numbers for NL4.3 and HXB-2 are U26942 and K03455, respectively.

## Abbreviations

3TC: lamivudine
AIC: Akaike Information Criterion
AZT: zidovudine
AZT-MP: AZT-monophosphate
AZT-TP: AZT-triphosphate
CI: confidence interval
EFV: efavirenz
HIV-1: human immunodeficiency virus type 1
IAS-USA: International AIDS Society-USA
NNRTI: non-nucleoside reverse transcriptase inhibitor
NRTI: nucleoside/nucleotide reverse transcriptase inhibitor
NVP: nevirapine
OR: odds ratio
PI: protease inhibitor
pVL: log10-transformed viral load
RNase H: ribonuclease H
RT: reverse transcriptase
RTI: RT inhibitor
TAM: thymidine analogue mutation
T/P: template/primer
WT: wild-type
